# Supplementary material for: SerpinA3K Deficiency Reduces Oxidative Stress in Acute Kidney Injury
Source: Int J Mol Sci. 2023 Apr 25;24(9):7815. doi: 10.3390/ijms24097815 (PMC10177890; doi:10.3390/ijms24097815)

Supplementary table S1

Modified Acute Tubular Necrosis Score
McLarnon SR, et al. JASN. 2022

Item	Description	Score
Loss of BB	PT without PAS+ apical membrane and increased luminal space.	0-4
Tubular dilation	Tubules with simplification and significant increase of luminal space.	0-4
Vacuolar degeneration*	Tubules with cytoplasmic inclusions of variable sizes, accompanied or not by pyknosis	0-4
Intratubular cast	Tubules with hyaline casts	0-4
Necrosis	Tubules with karyorrhexis and karyolysis. Also, denuded tubules.	0-4

0-4: refers to tubular area
0: no injury; 1: <25%; 2: <50%; 3: <75%; 4: 75-100%

*vacuolar degeneration is used instead of only vacuolization as male mice can exhibit normal degree of vacuolation. Vacuolar degeneration is usually accompanied by pyknosis in ischemic injury, therefore it is easier to score in this images.

Urinary SerpinA3K

uSerpinA3K

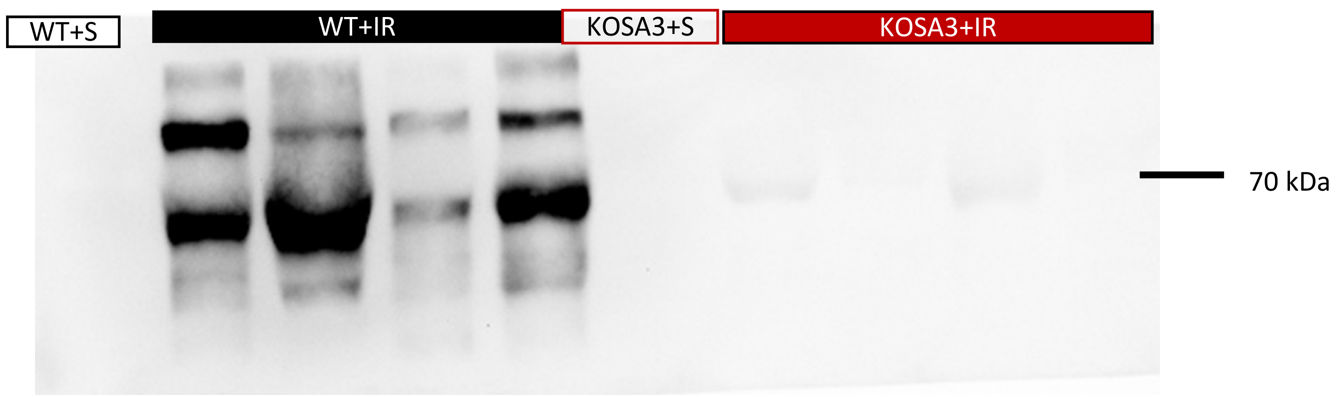

uSerpinA3K

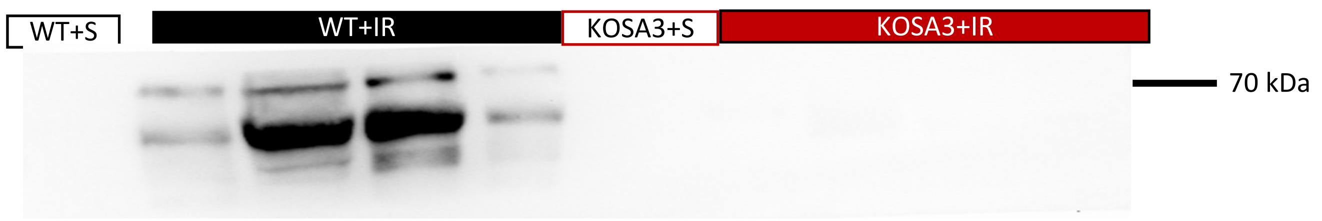

uSerpinA3K

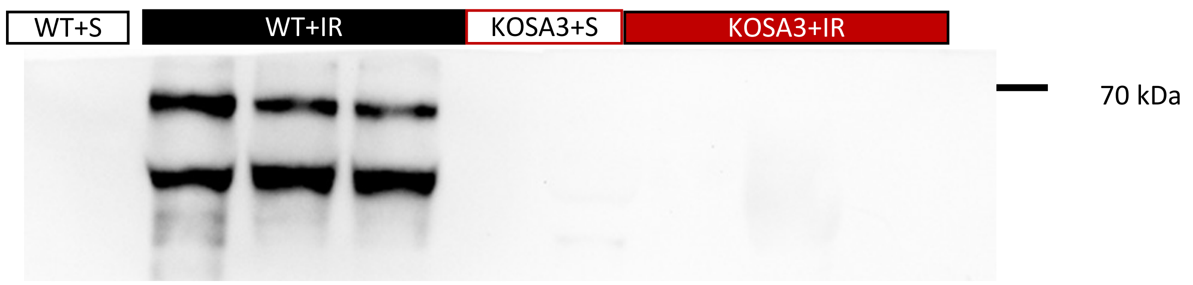

3 µl of urine

Primary antibody:
1:2,000 (anti-SerpinA3K; Proteintech, Cat. No. 554480-1-AO)
Secondary antibody:
1:2,500 (anti-Rb; Sigma, A0545)

Exposure time:
SerpinA3K: 5s

Uncropped gels

Urinary KIM-1

uKIM-1

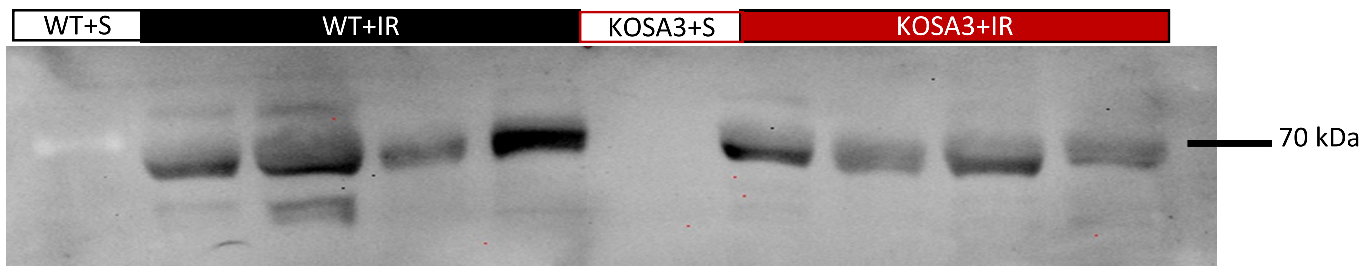

uKIM-1

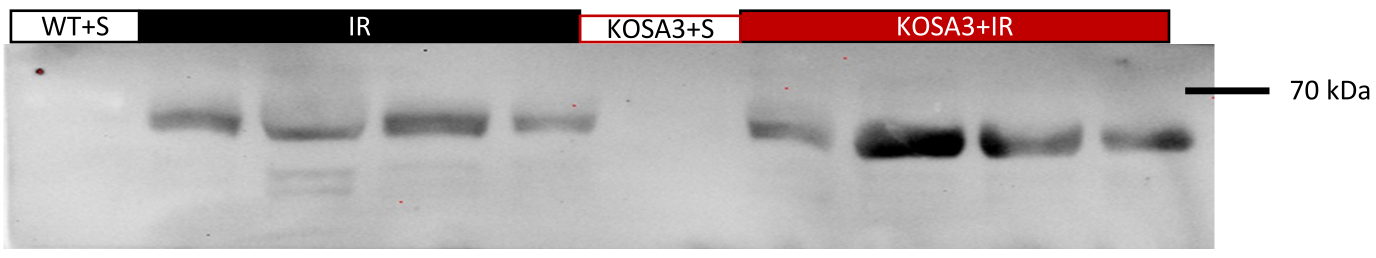

uKIM-1

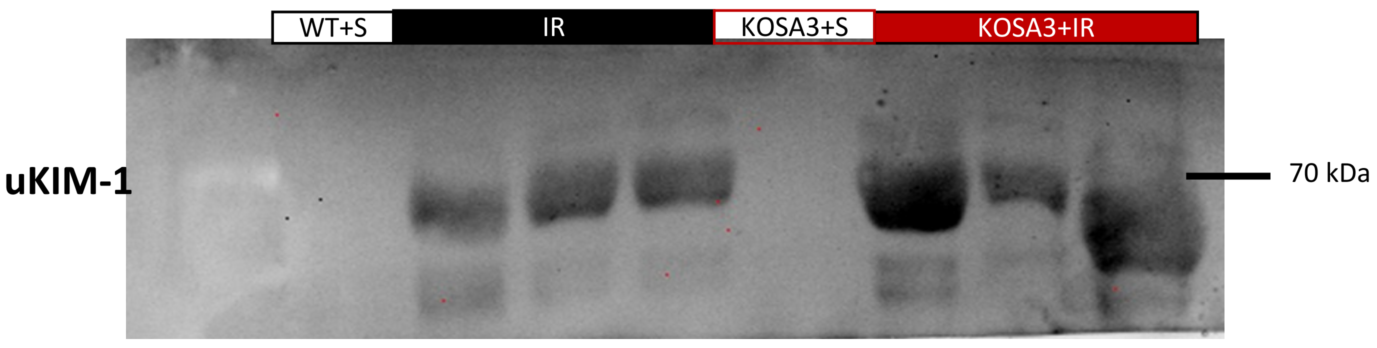

3 µl of urine

Primary antibody:
1:1,000 (anti-KIM-1; Boster, Cat. No. PA1632)
Secondary antibody:
1:2,500 (anti-Rb; Sigma, A0545)

Exposure time:
KIM-1: 1 min

Uncropped gels

Sirtuin 1

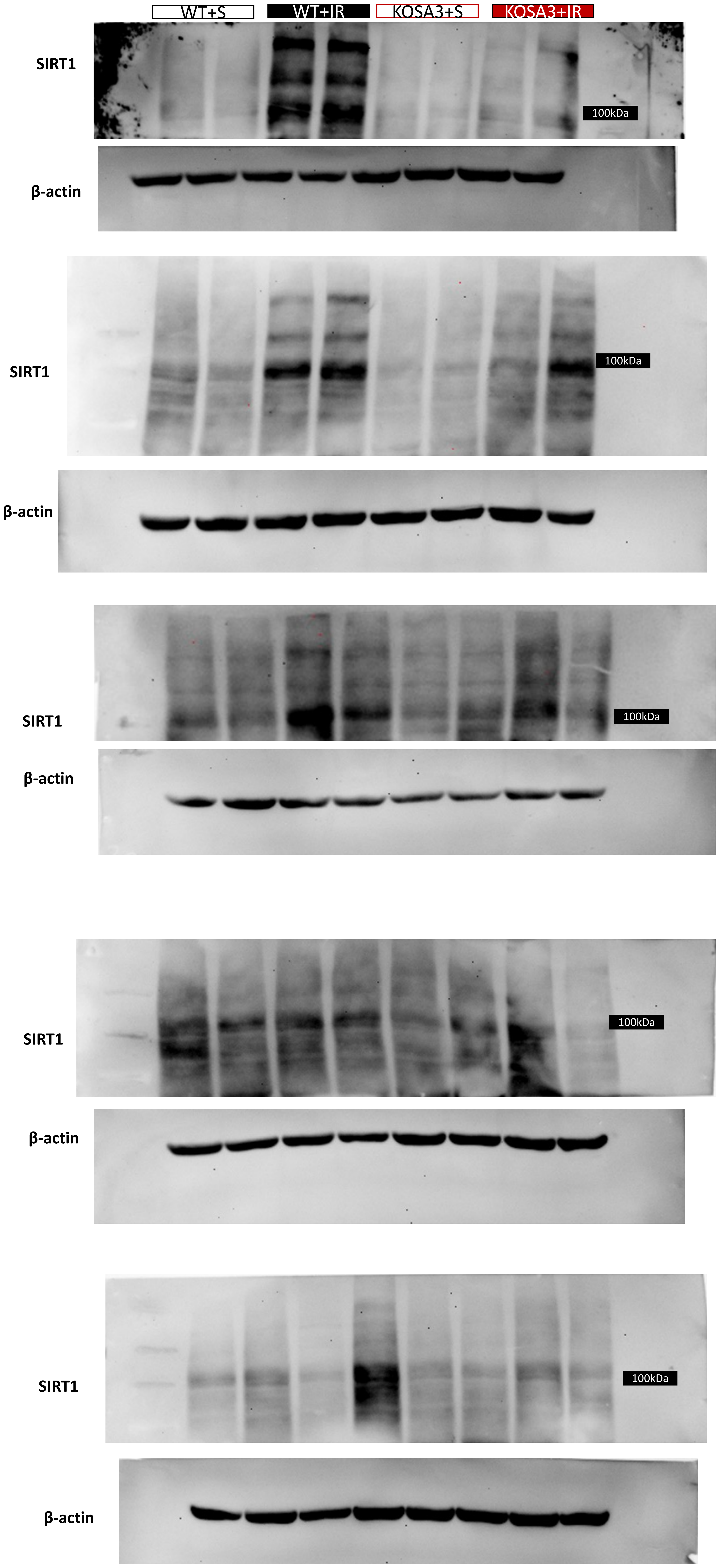

Renal cortex: 50 µg
Primary antibody:
1:1,500 (anti-SIRT) ; Santa Cruz, Cat. No. 74504
Secondary antibody:
1:1,500 (anti-mouse; Jackson, 115-035-174)

1:1,000,000 (HRP β-actina; Abcam, Cat. No. ab49900)

Exposure time:
SIRT1: 1 min
β-actina: 2 min

Uncropped gels

FOXO3

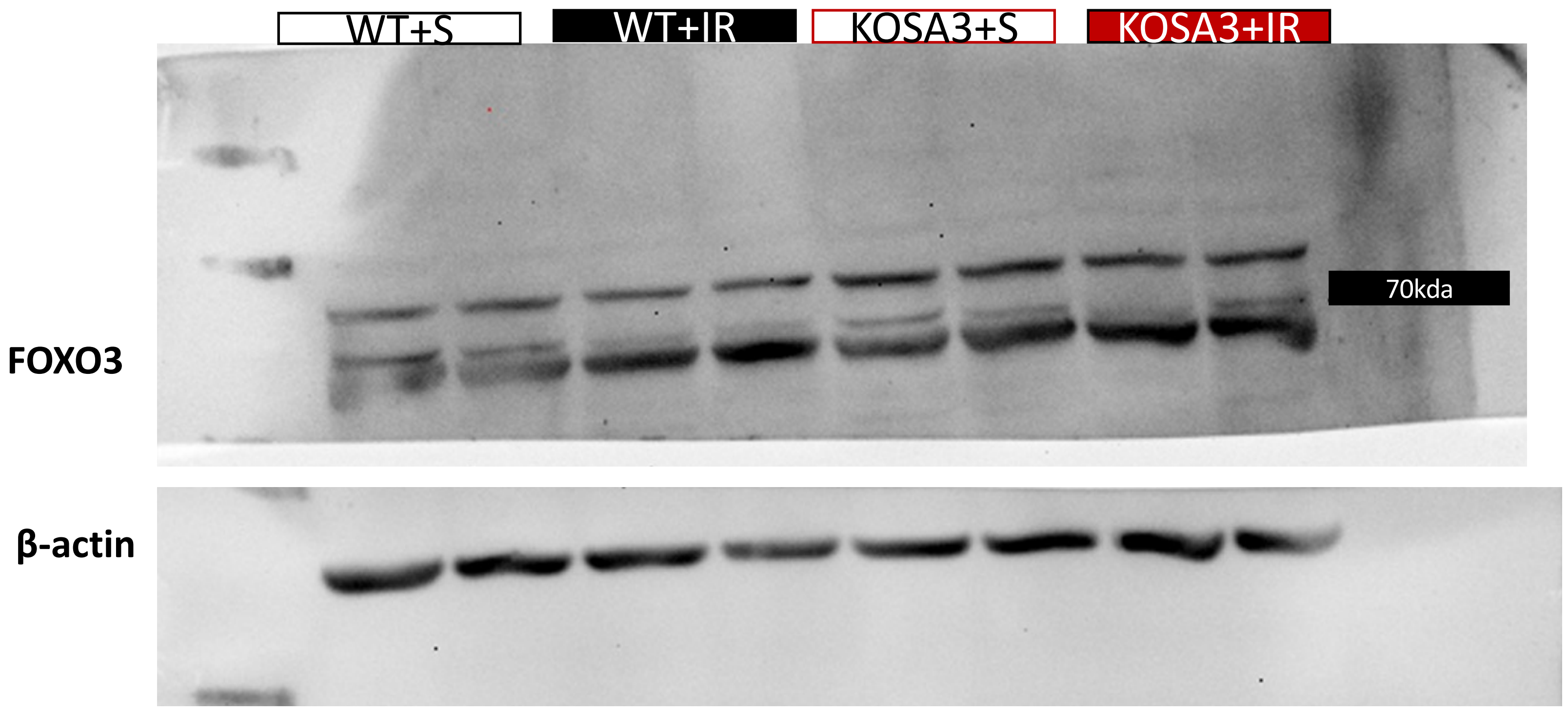

Renal cortex: 50 µg

Primary antibody:
1:2,000 (anti-FOXO3) ; Santa Cruz, Cat. No. sc-11351
Secondary antibody:
1:5,000 (anti-Rb; Sigma, A0545)

1:1,000,000 (HRP β-actina; Abcam, Cat. No. ab49900)

Exposure time:
FOXO3: 30s
β-actina: 1 min

Uncropped gels

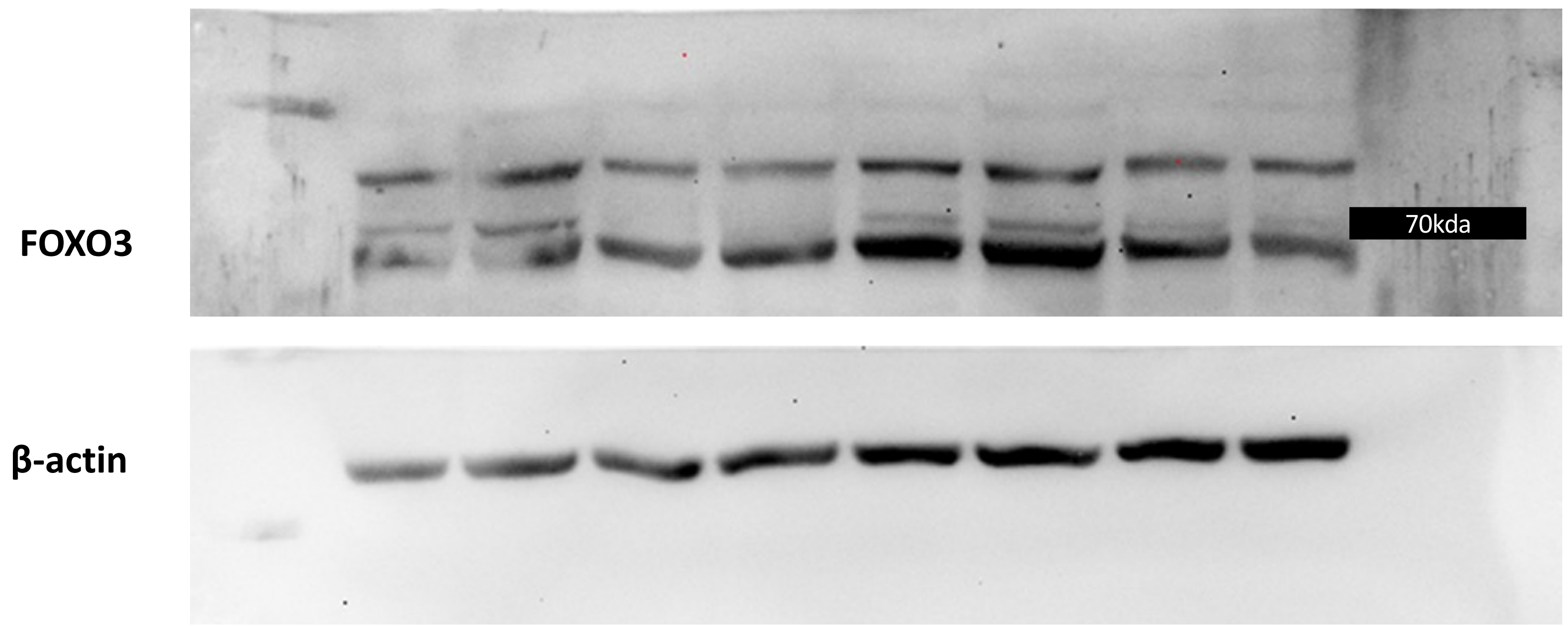

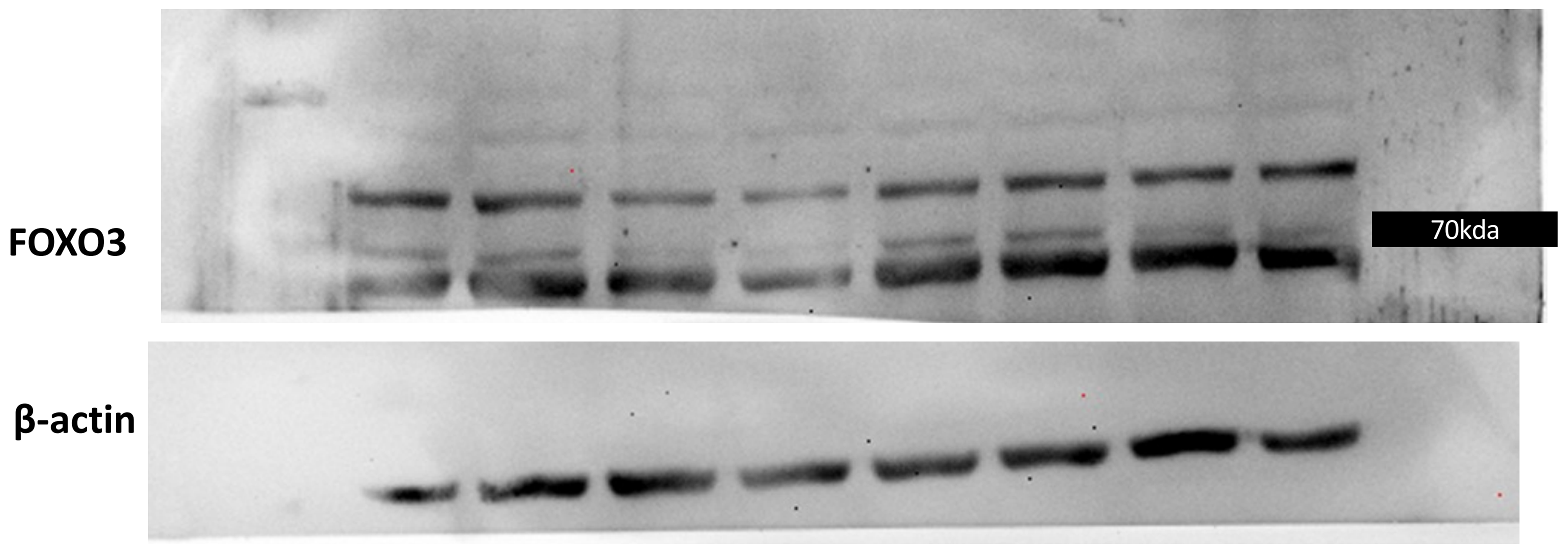

PGC-1 α

Renal cortex: 50 µg

Primary antibody:
1:2,000 (anti-PGC-1α) ; Invitrogen, Cat. No. PA5-38021
Secondary antibody:
1:5,000 (anti-Rb; Sigma, A0545)

1:1,000,000 (HRP β-actina; Abcam, Cat. No. ab49900)

Exposure time:
FOXO3: 1min
β-actina: 1 min

Uncropped gels

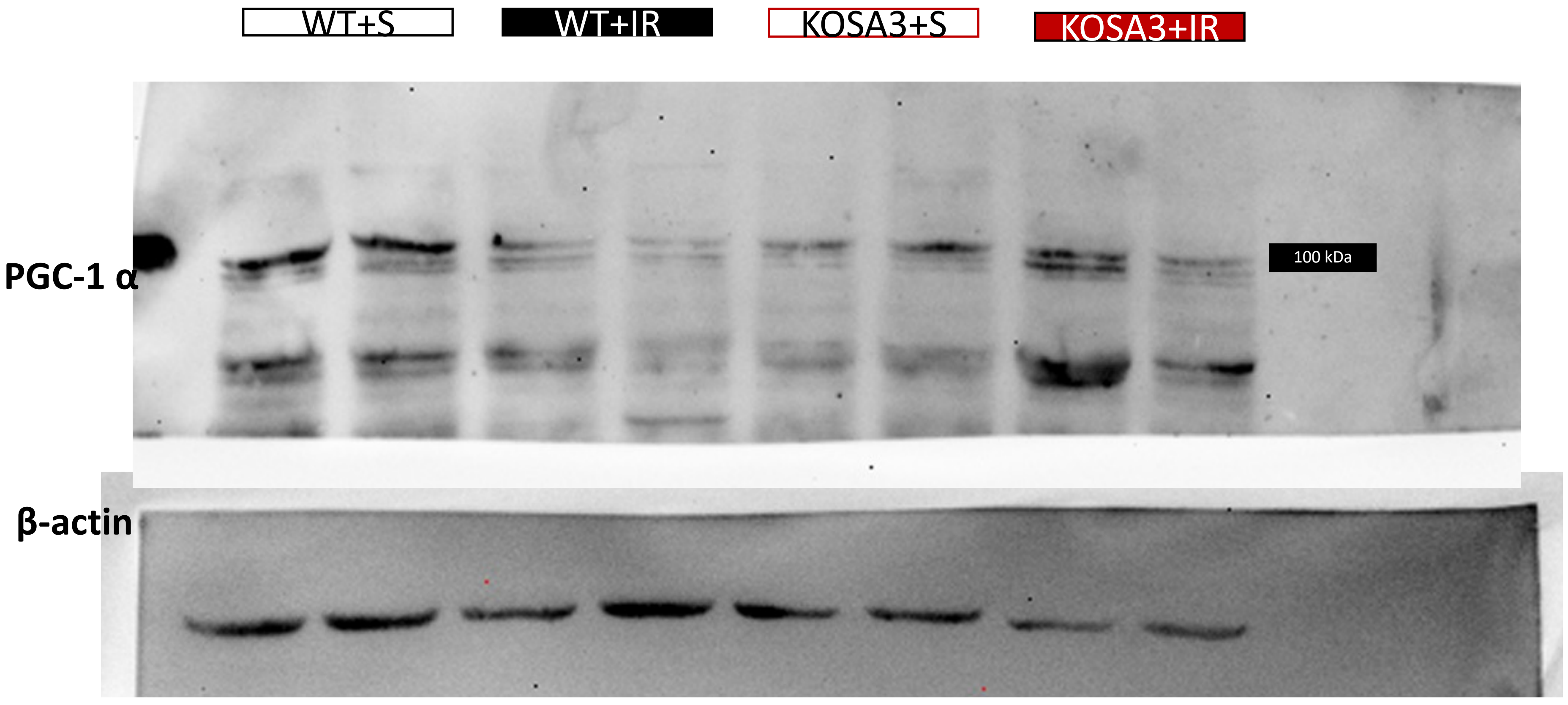

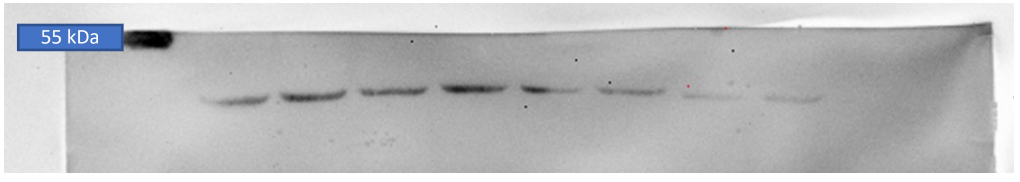

Beta-actin affected by control marks

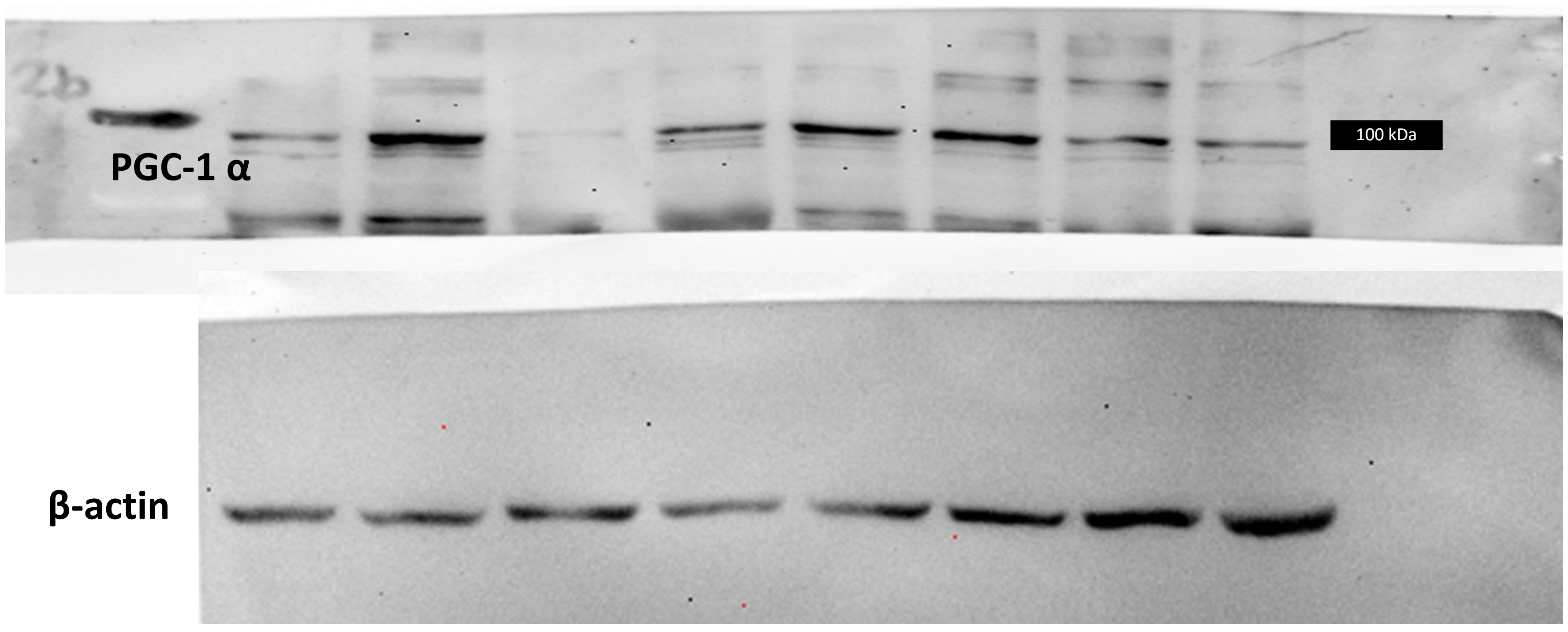

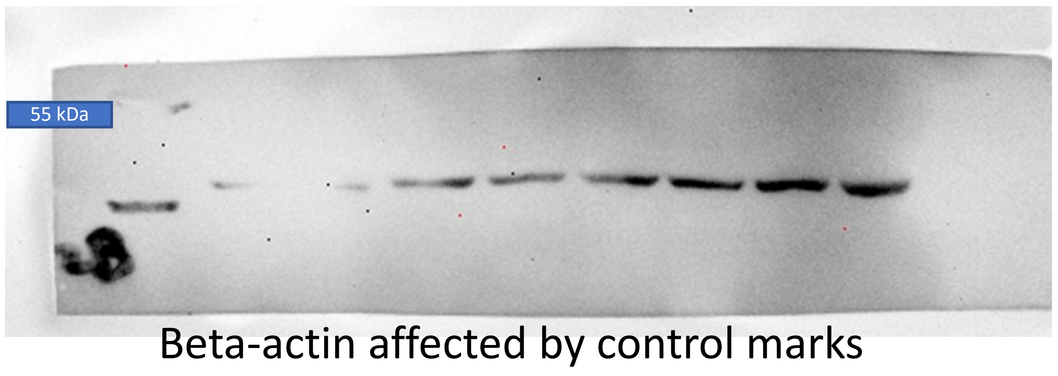

Beta-actin affected by control marks

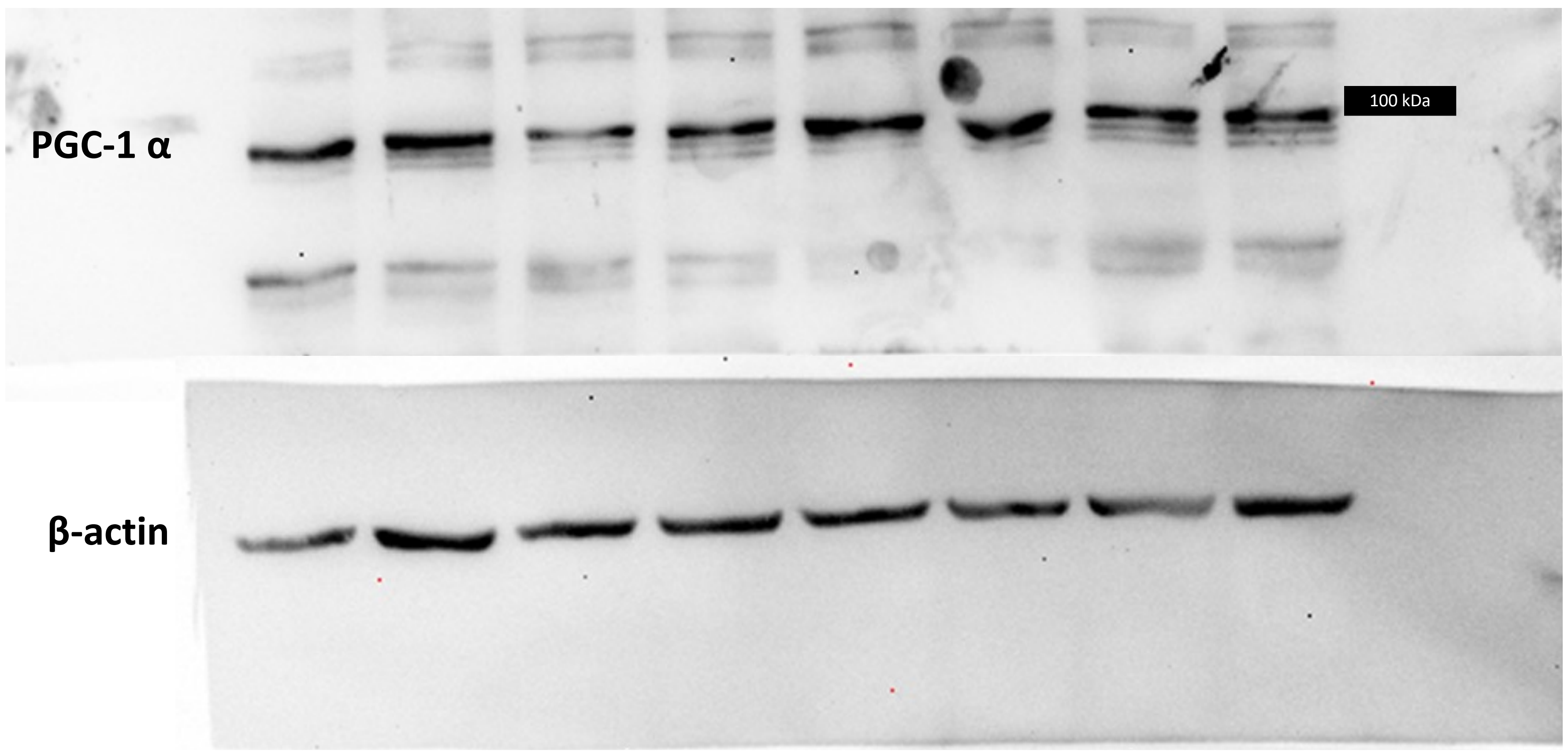

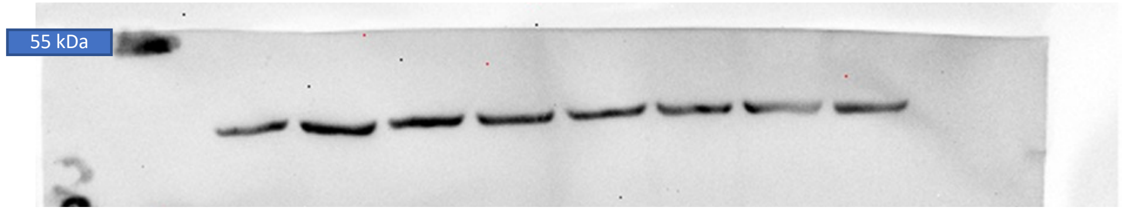

Beta-actin affected by control marks

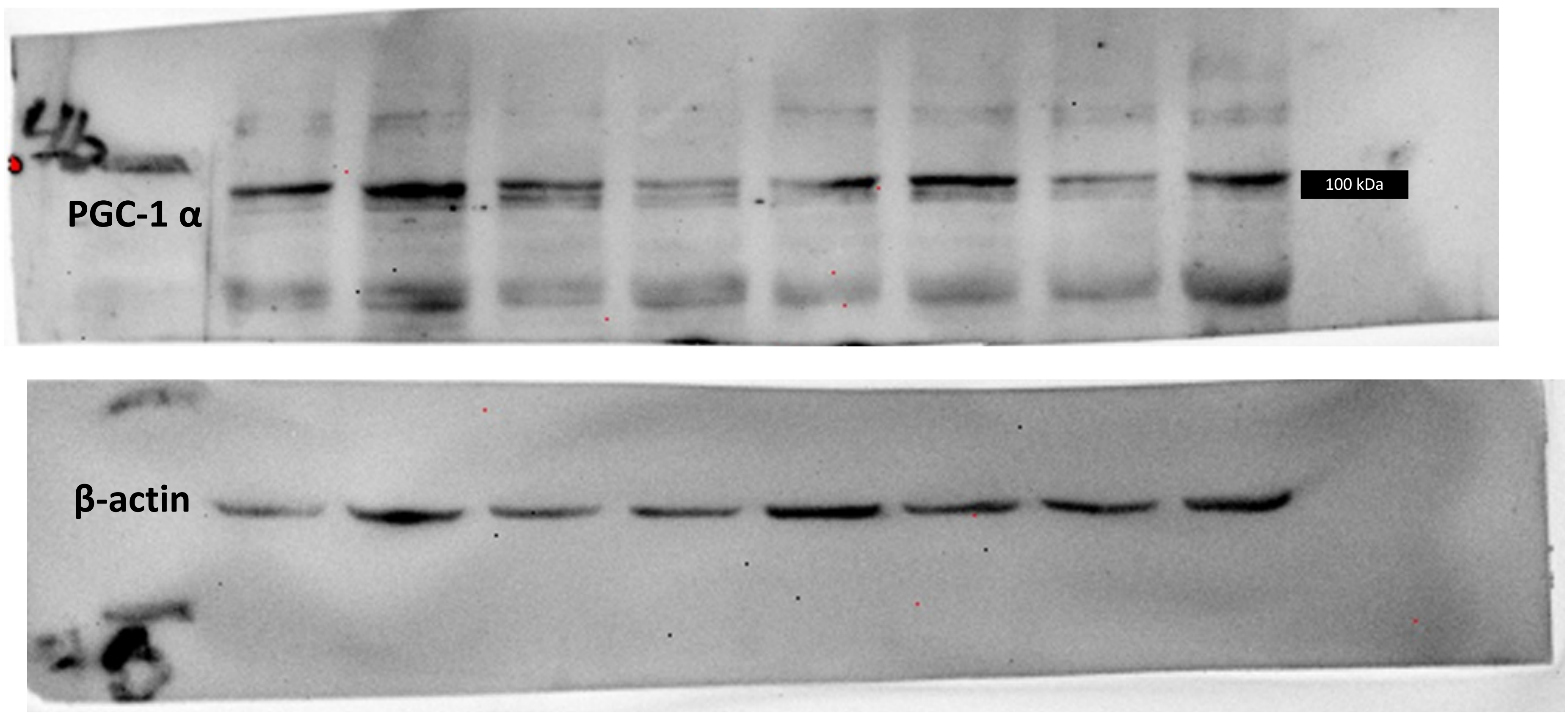

BAX

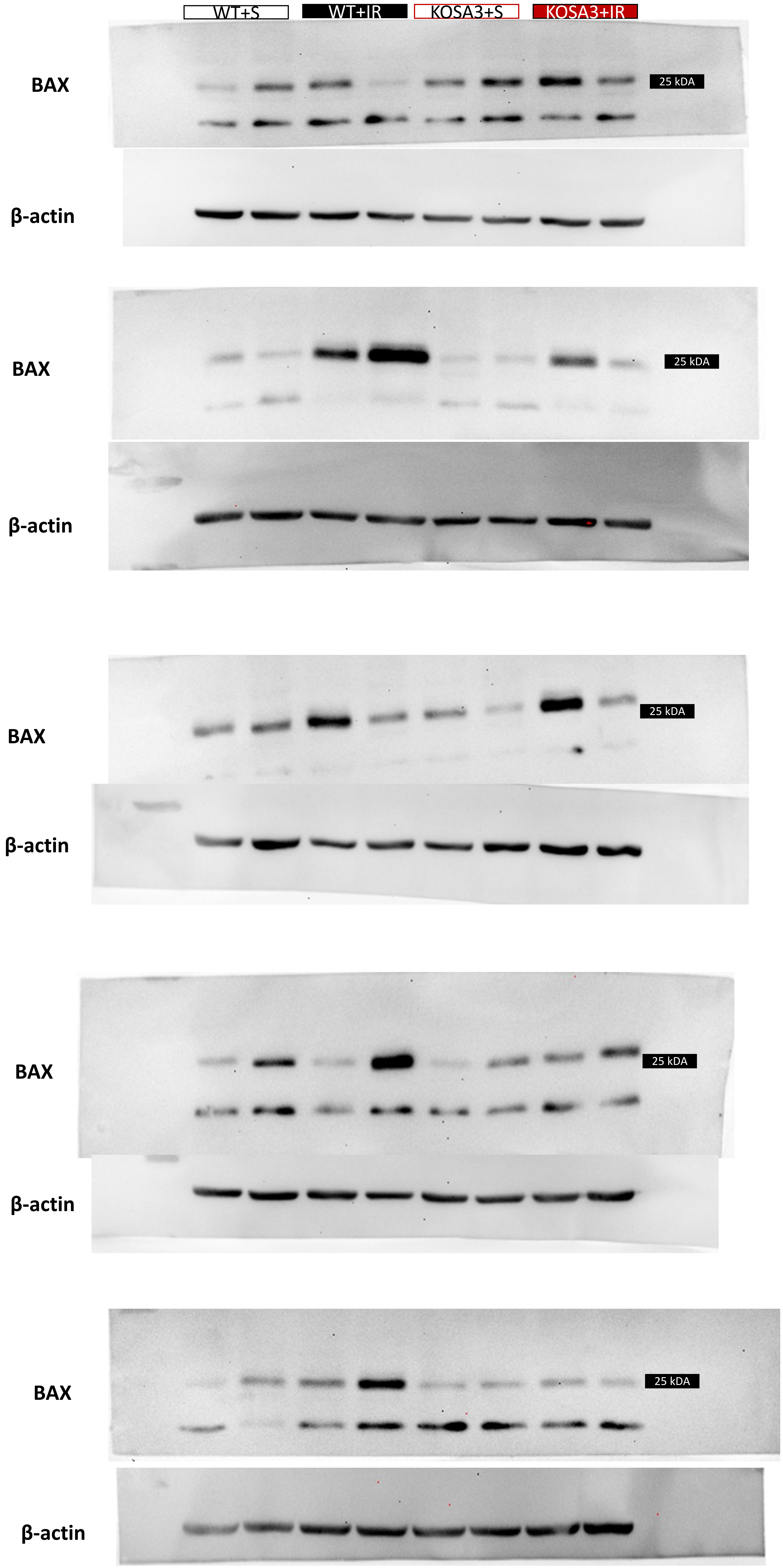

Renal cortex: 50 μg
Primary antibody:
1:2,000 (anti-BAX) ; Sigma-Aldrich, Cat. No. SAB5701333
Secondary antibody:
1:5,000 (anti-Rb; Sigma, A0545)

1:1,000,000 (HRP β-actina; Abcam, Cat. No. ab49900)

Exposure time:
BAX: 15s
β-actina: 1 min

Uncropped gels

β-catenin

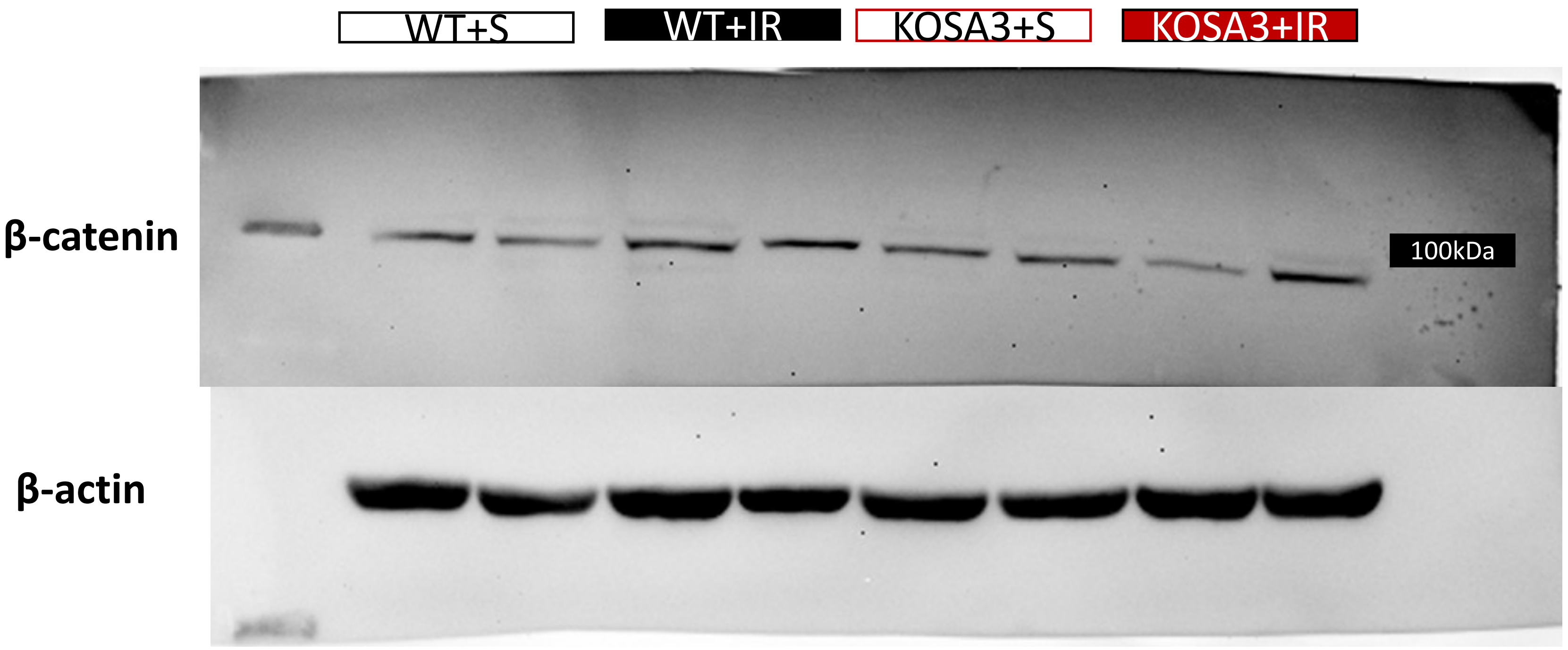

Renal cortex: 75 µg

Primary antibody:
1:1,000 (anti-β-catenin) ; Cell signaling, Cat. No. 8480S
Secondary antibody:
1:10,000 (anti-Rb; Sigma, A0545)

1:1,000,000 (HRP β-actina; Abcam, Cat. No. ab49900)

Exposure time:
β-catenin: 3 min
β-actina: 2 min

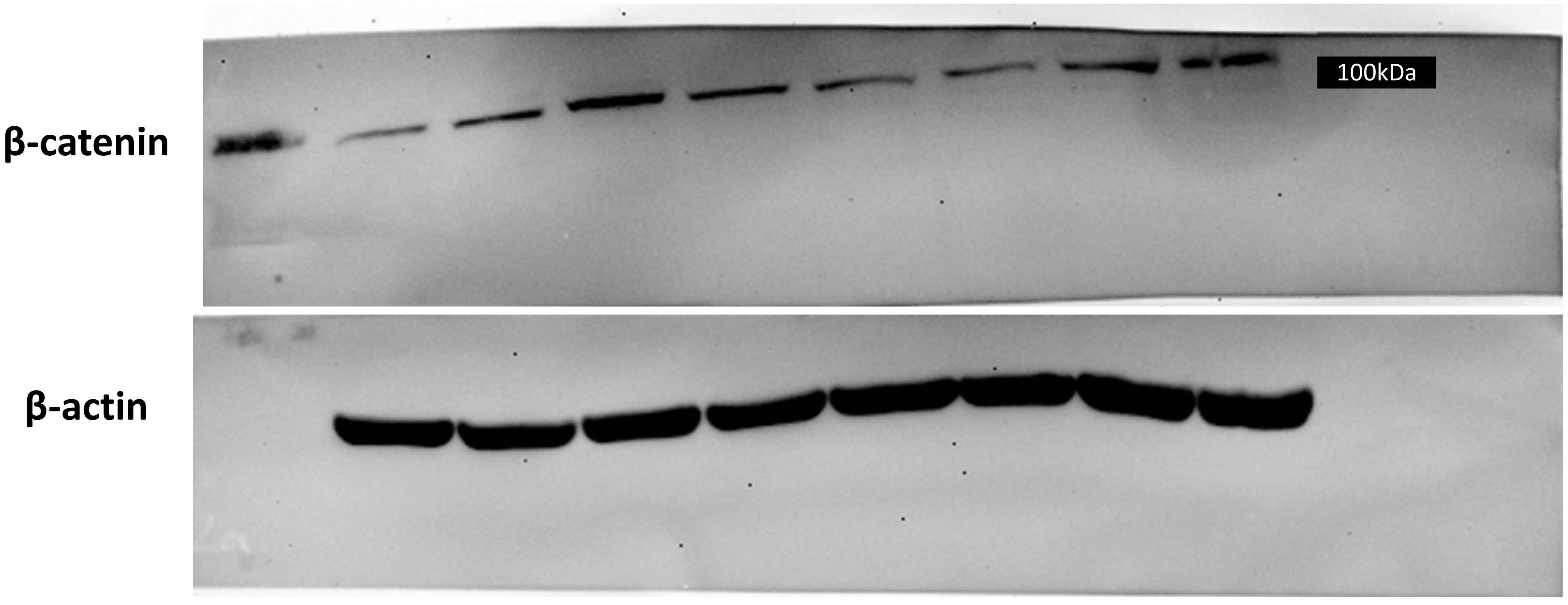

Uncropped gels

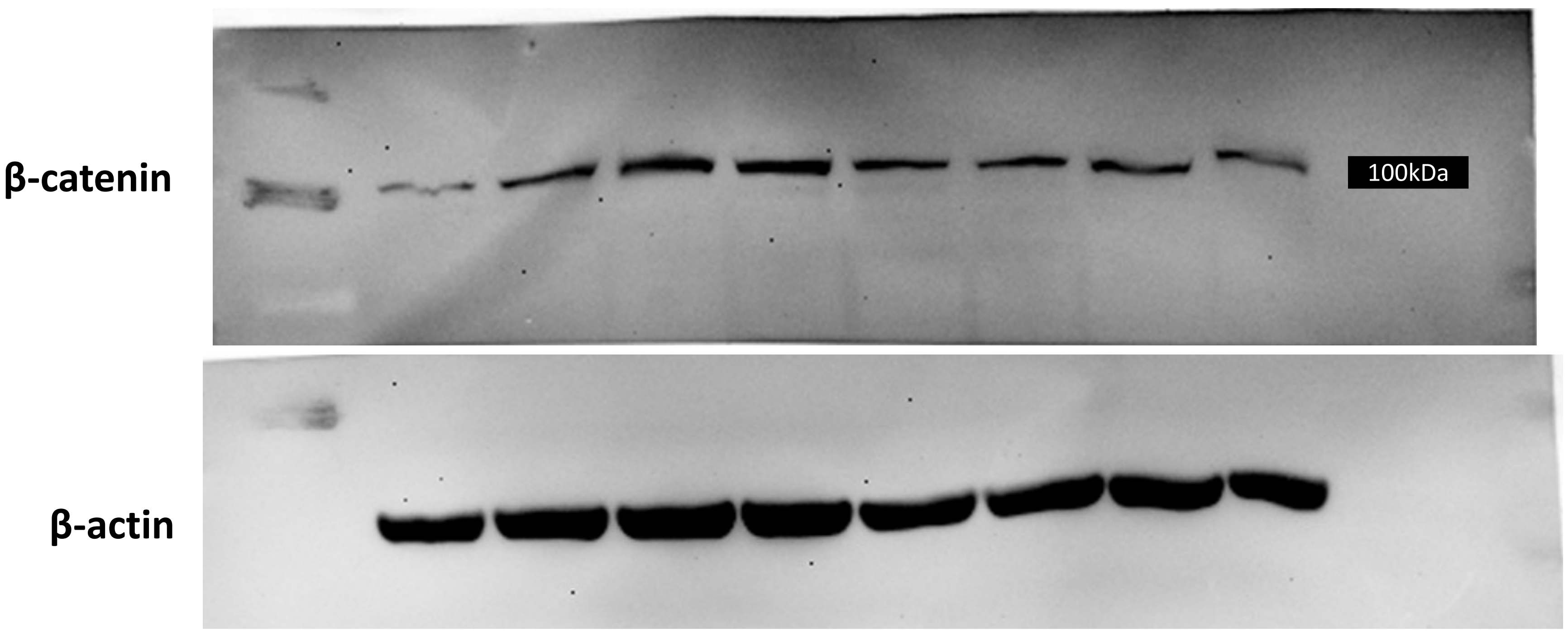

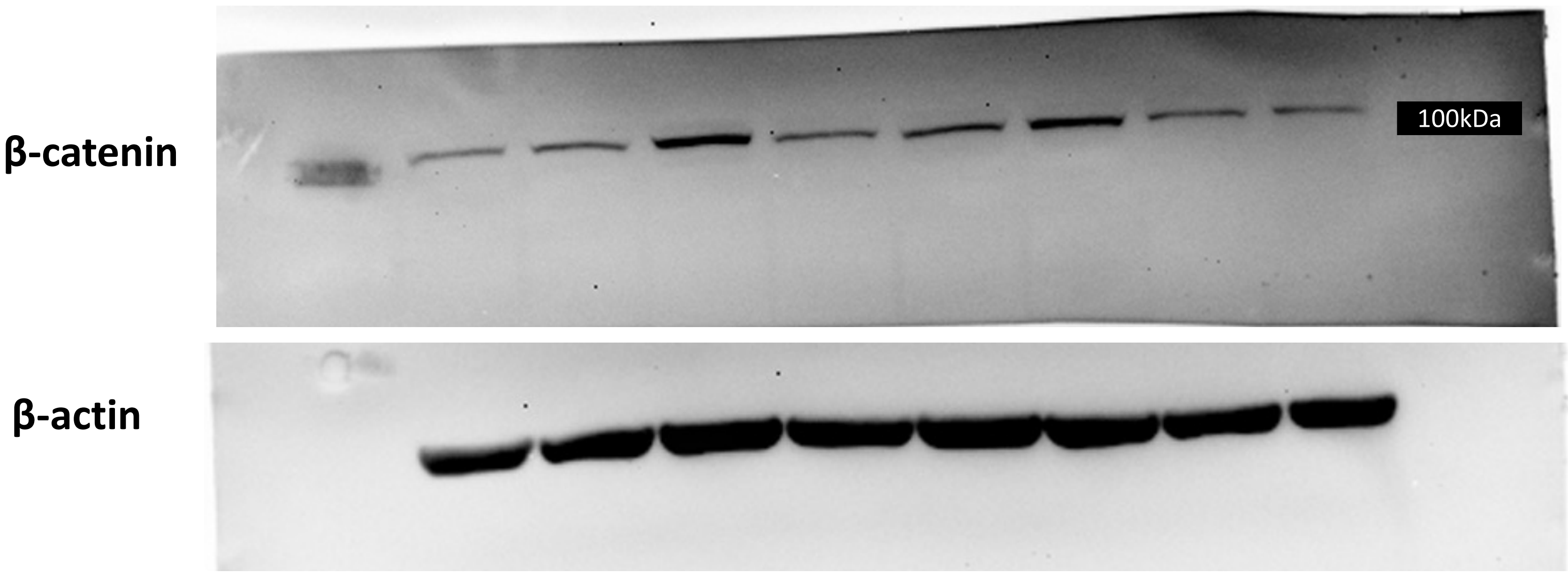

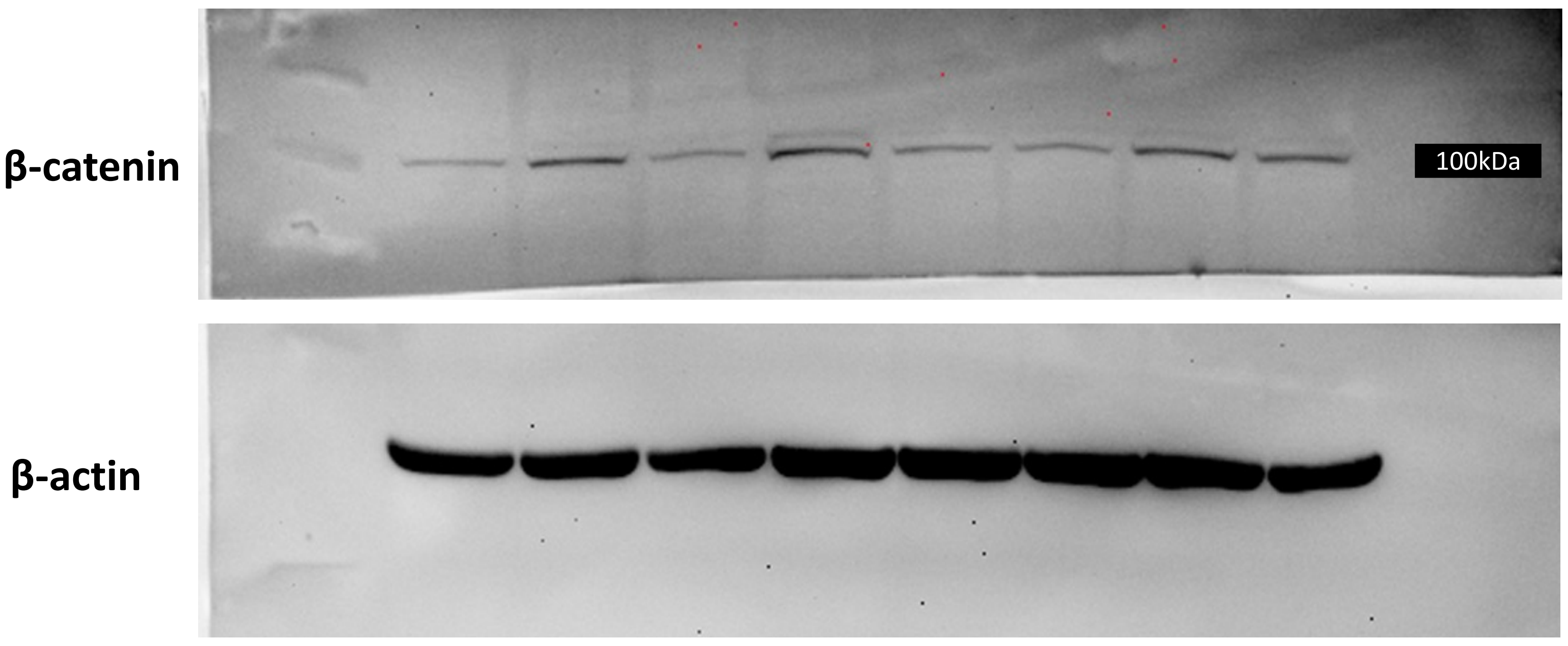

Supplement: Supplementary file 1 [file ijms-24-07815-s001.zip › ijms-2327822-supplementary.pdf]
